# Categorical Biases in Perceiving Spatial Relations

**DOI:** 10.1371/journal.pone.0098604

**Published:** 2014-05-28

**Authors:** Alexander Kranjec, Gary Lupyan, Anjan Chatterjee

**Affiliations:** 1 Psychology Department, Duquesne University, Pittsburgh, Pennsylvania, United States of America; 2 Center for the Neural Basis of Cognition, Carnegie Mellon University, Pittsburgh, Pennsylvania, United States of America; 3 Department of Psychology, University of Wisconsin, Madison, Wisconsin, United States of America; 4 Center for Cognitive Neuroscience, University of Pennsylvania, Philadelphia, Pennsylvania, United States of America; Goldsmiths, University of London, United Kingdom

## Abstract

We investigate the effect of spatial categories on visual perception. In three experiments, participants made same/different judgments on pairs of simultaneously presented dot-cross configurations. For *different* trials, the position of the dot within each cross could differ with respect to either categorical spatial relations (the dots occupied different quadrants) or coordinate spatial relations (the dots occupied different positions within the same quadrant). The dot-cross configurations also varied in how readily the dot position could be lexicalized. In harder-to-name trials, crosses formed a “+” shape such that each quadrant was associated with two discrete lexicalized spatial categories (e.g., “above” and “left”). In easier-to-name trials, both crosses were rotated 45° to form an “×” shape such that quadrants were unambiguously associated with a single lexicalized spatial category (e.g., “above” or “left”). In Experiment 1, participants were more accurate when discriminating categorical information between easier-to-name categories and more accurate at discriminating coordinate spatial information within harder-to-name categories. Subsequent experiments attempted to down-regulate or up-regulate the involvement of language in task performance. Results from Experiment 2 (verbal interference) and Experiment 3 (verbal training) suggest that the observed spatial relation type-by-nameability interaction is resistant to online language manipulations previously shown to affect color and object-based perceptual processing. The results across all three experiments suggest that robust biases in the visual perception of spatial relations correlate with patterns of lexicalization, but do not appear to be modulated by language online.

## Introduction

Do spatial categories like “left” and “above” penetrate perception? Although it has been demonstrated that space can influence how we perceive other domains as varied as time [1], and soccer [2], there is surprisingly little research investigating whether spatial perception is modulated by spatial categories. Studies with infants have previously investigated how patterns of discriminating more or less typical spatial categories develop [3], [4], [5]. However, the limitations of infant research (e.g., the reliance on habituation and preferential looking paradigms) make it difficult to distinguish between effects of spatial categories on perception versus effects of categories on memory. Similarly, studies with adults investigating relations between spatial categorization and visual processing generally have not examined the influence of spatial categories on perception. Instead they have tended to probe how verbal labels [6], verbal interference [7], [8], [9], conceptual heuristics [10], [11], or cross cultural differences in frames of reference [12], [13] influence spatial memory using recall, physical reconstruction, reorientation, or rating tasks.

To directly test the effect of spatial categories on visual perception the present study makes use of a well-known neuropsychological distinction. Kosslyn [14] originally proposed that spatial relations can be divided into two broad types: *Categorical* relations refer to discrete spatial relations frequently lexicalized by locative prepositions like “left”, “right”, “above”, and “below.” *Coordinate* relations are finer-grained metric relations, such as analog distances. Categorical representations specify abstract, equivalent classes of spatial relations, whereas coordinate representations specify the exact locations of objects in space: information critical for reaching and navigation [14], [15], [16], [17]. Originally, Kosslyn [14] speculated that cortical specializations for categorical spatial information processing in the left hemisphere, and coordinate information in the right, was the evolutionary result of prior hemispheric specializations for speech. That is, the functional and anatomical relation between categorical spatial processing and language began with language—language providing the “seed” for spatial information processing of a complimentary type in nearby left hemisphere anatomical structures. Later, Kosslyn [18] more or less reversed his theory, hypothesizing that low-level perceptual biases in left hemisphere structures important for abstraction (and categorization) served as a precursor for the development of language in proximate cortical areas. (See [19] for a short review.)

We were interested in whether the discrimination of categorical and coordinate information differs as a function of the meaning of particular spatial relations: Specifically, we predicted that categorical perceptual discrimination would be easier between categories discretely lexicalized by common locative prepositions than for categories less easily (or more ambiguously) lexicalized. That is, discriminating between two perceptually different spatial relations that are discretely lexicalized (*above* vs. *left*) should be *easier* than discriminating between two perceptually different spatial relations that share a common spatial category between them (*above-left* vs. *below-left*). Conversely, insofar as lexicalized distinctions are associated with more common categories [20], [21], [22], it should be *more difficult* to make coordinate judgments when discriminating between two perceptually different spatial relations within easier-to-name spatial categories compared to more ambiguously lexicalized categories. In short, we predict that “nameability” will have opposite effects on categorical and coordinate discriminations. Better nameability will make locations *between* spatial categories more distinctive, while making locations *within* spatial categories more alike. (See [23] for further rationale and additional empirical work regarding this prediction.)

## Experiment 1

### 1.1. Methods

#### 1.1.1 Participants

Ten adults participated for pay or course credit (6 women; median age  = 24). Participants in all experiments were right-handed, native English speakers and gave written informed consent. The Institutional Review Board of the University of Pennsylvania approved all experimental protocols.

#### 1.1.2. Procedure and Materials

**Figure 1:**
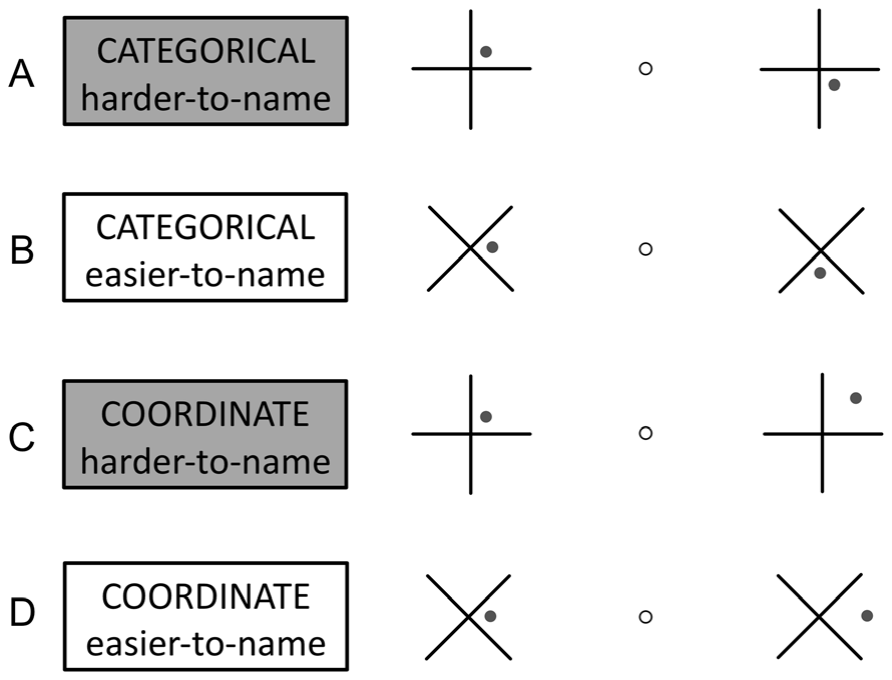
Examples of the four types of “different” trials. Two dot-cross grids were displayed simultaneously to the left and right of central fixation. In the actual experiments, the dot was red and appeared an equal number of times in each of the 4 quadrants across all trials.

Participants made same/different judgments on pairs of dot-cross grids presented simultaneously for 200 ms to the left and right of central fixation (Figure 1; Stimuli were adapted from a previous study [24]). The participants' task was to respond “same” (via keypress) anytime the two configurations were identical and respond “different” otherwise. The critical “different” trials, shown in Figure 1, varied on two variables of interest: relation type (categorical or coordinate), and nameability (easier-to-name or harder-to-name).

**Figure 2:**
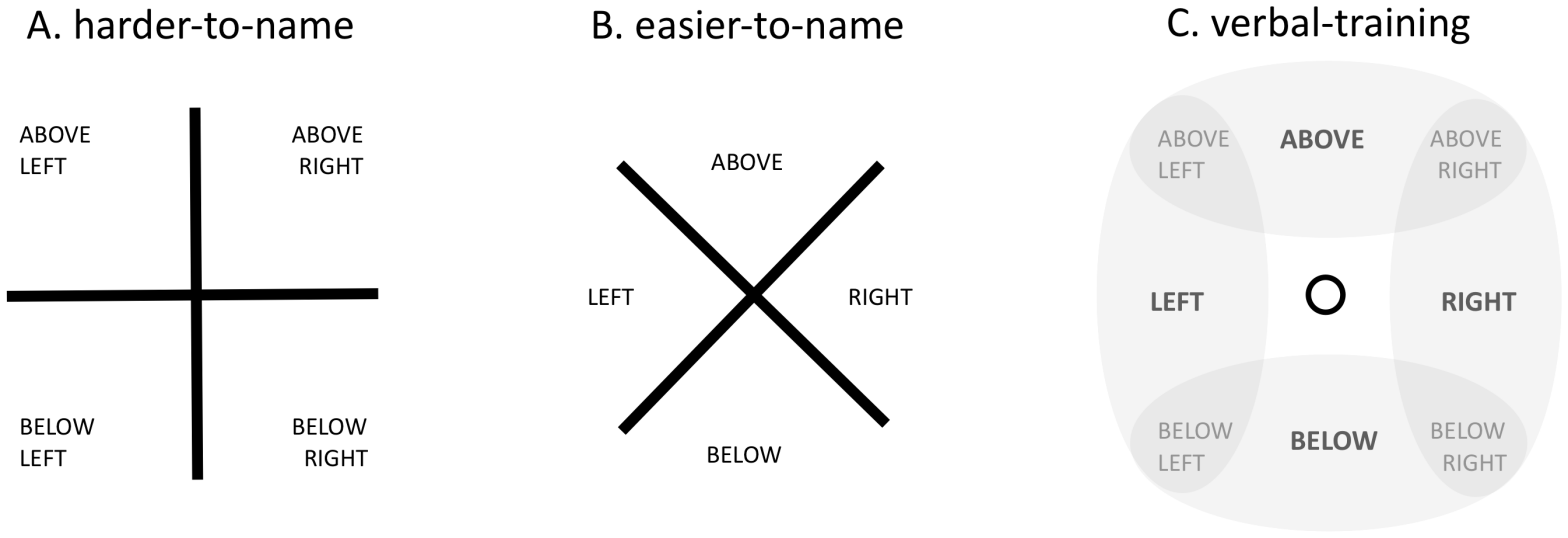
Harder vs. easier-to-name spatial categories. (A) In *harder-to-name* trials each quadrant was associated with two spatial prepositions (e.g. *above* AND *left*). (B) In *easier-to-name* trials each quadrant was unambiguously associated with a discrete spatial preposition (e.g. *above* OR *left*). (C) For *verbal training* in Experiment 3, each of the four positions corresponding to the “×” partition (not shown during training) was valid for a single label (*above*, *below*, *left*, or *right*). Each of the four positions corresponding to the “+” partition was valid for two labels (*above* and *right*, *above* and *left*, *below* and *right*, or *below* and *left*).

On *categorical* trials the dots occupied different quadrants, but maintained the same distance (15, 30, 45, or 60 pixels) from the origin in each grid (displayed to the left and right of fixation). On *coordinate* trials, the dots occupied the same quadrant, but were located at various distances from the origin (also 15, 30, 45, or 60 pixels). Nameability was instantiated by a simple rotation of the display. In *harder-to-name* trials, crosses were composed of intersecting vertical and horizontal lines forming a “+” such that each quadrant was associated with two spatial prepositions (e.g. *above* AND *right*). In *easier-to-name* trials, both crosses were rotated 45° to form an “×” such that each quadrant was unambiguously associated with a discrete spatial preposition (e.g. *below* OR *right*). (See Figure 2A and 2B.) [Performance on training trials in Experiment 3 confirmed that the quadrants in “×” displays depicted more prototypical spatial categories compared to “+” displays. (See Figure 2C and Section 3.2.)]

All trial-types were intermixed. Each trial consisted of a 500 ms pre-fixation delay, followed by a 250 ms fixation cross and 200 ms stimulus presentation. The experiment was self-paced in that a new trial was triggered upon making a response to the previous one. There were a total of 512 trials; 50% of trials were *different* trials, 50% were *same* trials. The 256 *different* trials consisted of equal numbers of the four main trial types. (See Figure. 1).

### 1.2. Results

The analyses of all experiments include only the critical “different” trials because only these trials differ on the two variables of interest: spatial relation type and nameability. We hypothesized that categories unambiguously lexicalized by the words “left”, “right”, “above”, and “below”, correspond to more discrete spatial categories and should therefore lead to more accurate detection of between-category differences. Conversely, because coordinate discriminations require discrimination *within* a category, when making coordinate judgments the pattern should reverse and performance should be *worse* for easier-to-name trials relative to harder-to-name categories.

**Figure 3:**
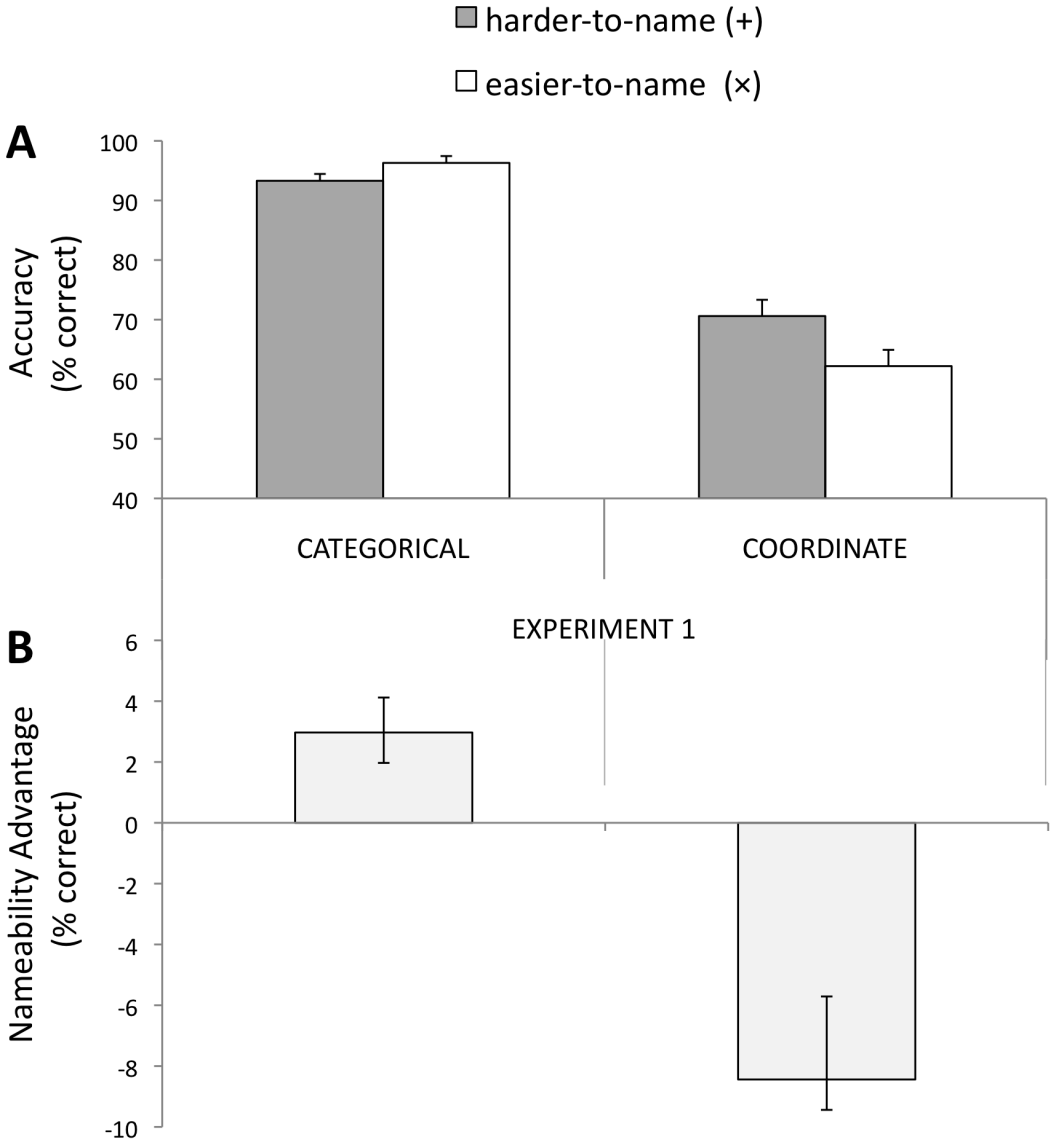
Accuracy results for Experiment 1. (A) Overall % accuracy for all different trials. (B) Nameability advantage (easier-to-name trials minus harder-to-name trials). A positive difference score indicates an advantage on easier-to-name trials; a negative score indicates a disadvantage on easier-to-name trials. Error bars represent 1SE of the mean difference between easier-to-name and harder-to-name trials.

**Accuracy**. As evident from Figure 3, there was a significant main effect of spatial relation type; overall participants were more accurate on categorical as compared to coordinate trials, *F*(1,9) = 258.88, *p*<.001. The main effect of nameability was not significant. The critical nameability (harder-to-name vs. easier-to-name) × spatial relation-type (categorical vs. coordinate) interaction was highly reliable in the predicted direction, *F*(1,9) = 12.96, *p*<.01 (Fig. 3). Planned t-tests showed that for categorical (between-category) trials, direct correspondence with easier-to-name spatial positions improved performance by 3%, CI [−0.056, −0.004], *t*(9) = 2.58, *p* = .03. For coordinate (within-category) trials, easier-to-name positions decreased accuracy by 8.4%, CI [0.027, 0.146], *t*(9) = 3.09, *p* = .01.

**Reaction Time**. Analysis of RTs revealed a congruent pattern of results, with no evidence of a speed-accuracy tradeoff. (*M*+_CAT_  = 599 ms, *M*×_CAT_  = 552 ms, *M*+_COOR_  = 624, *M*×_COOR_  = 640 ms). There was a main effect of spatial relation-type showing a highly reliable advantage for categorical trials, *F*(1,9) = 13.60, *p* = .005, and a main effect of nameability showing a slight advantage for easier-to-name trials, *F*(1,9) = 5.94, *p* = .038. As with accuracy, the pattern of RTs showed a reliable nameability × spatial relation-type interaction, *F*(1,9) = 10.28, *p*<.01.

Planned comparisons show that for categorical relations, participants responded faster to easier-to-name trials by 47 ms, CI [27.43, 66.62], t(9) = 5.43, *p*<.0005. For coordinate relations, RTs were faster on harder-to-name trials by 17 ms, CI [−48.50, 15.50], though this difference was non-reliable, t(9) = 1.17, *p*>.20.

### 1.3. Discussion

These results suggest that there is a relationship between discretely lexicalized spatial categories and perceptual processing of categorical and coordinate spatial relations. Even though the observed results were obtained without the explicit use of any particular category labels, it is possible that the perceptual processes involved in making the same-different decision are modulated, online, by implicitly activated verbal labels [21], [25].

## Experiment 2 (Verbal Versus Visual-Interference)

To investigate whether the online engagement of verbal representations is critical in producing the effects observed in Experiment 1, Experiment 2 contrasted performance on the original task under conditions of verbal and visual interference. Verbal interference is hypothesized to down-regulate the involvement of language in task performance [26], [27]. If language plays a critical role in the observed perceptual biases, the advantage on the categorical easier-to-name trials and coordinate harder-to-name trials (Exp. 1) should be attenuated by verbal interference. We predicted that if language plays a critical role in the observed perceptual biases, then down-regulating the activation of category labels via a verbal interference task should decrease the difference in performance between the easier-to-name (x) and harder-to-name (+) trials.

### 2.1. Methods

#### 2.1.1 Participants

Seventeen adults participated for pay or course credit (9 women; median age  = 22). All participants were right-handed, native English speakers.

#### 2.1.2. Procedure and Materials

Participants performed a same/different judgment task on pairs of dot-cross configurations (as in Experiment 1) with concurrent verbal or visual-interference tasks. The design of the interference tasks was based on that of a previous study investigating the role of language on color perception [28]. Both interference tasks in the present experiment used a one-back match paradigm. For the verbal-interference task, at the start of a 4 trial sequence of the same/different discrimination trials, one of 10 color words was presented for 1400 ms (beige, black, brown, gray, orange, pink, purple, violet, white, or yellow). Participants were instructed to remember the word during the subsequent discrimination trials. After 4 trials, another color word was presented at the beginning of the next trial sequence. Participants were told to press the space bar with their left hand if the color word matched the previous one. For the visual-interference task, participants had to instead remember one of 10 different fractal patterns, also presented for 1400 ms at the beginning of a 4 trial sequence. Interference type was manipulated within subjects. There were 128 trials per run, with 2 runs of verbal-interference and 2 runs of visual-interference conditions for 512 total trials. Interference-condition type alternated, and the order was counterbalanced. For both verbal and visual-interference conditions, 15% of word or pattern trials were “match trials” and therefore required a response on the interference task. The remaining 85% were “nonmatch trials” and did not require a response.

### 2.2. Results

**Performance on Interference Tasks**. Participants made more errors on the visual match/nonmatch task (match trials  = 86.6% correct, different trials  = 93.8% correct) than the verbal task (match trials  = 92.9% correct; different trials  = 95.9% correct); main effect of interference type, *F* [1], [16] = 4.78, *p* = .044. There is therefore some reason to believe that the visual-interference task was more difficult than the verbal-interference task, particularly for the 15% of trials requiring a match response; response type (match vs. nonmatch) × interference-type (verbal vs. visual) [*F*(1,16) = 16.30, *p*<.001].

**Figure 4:**
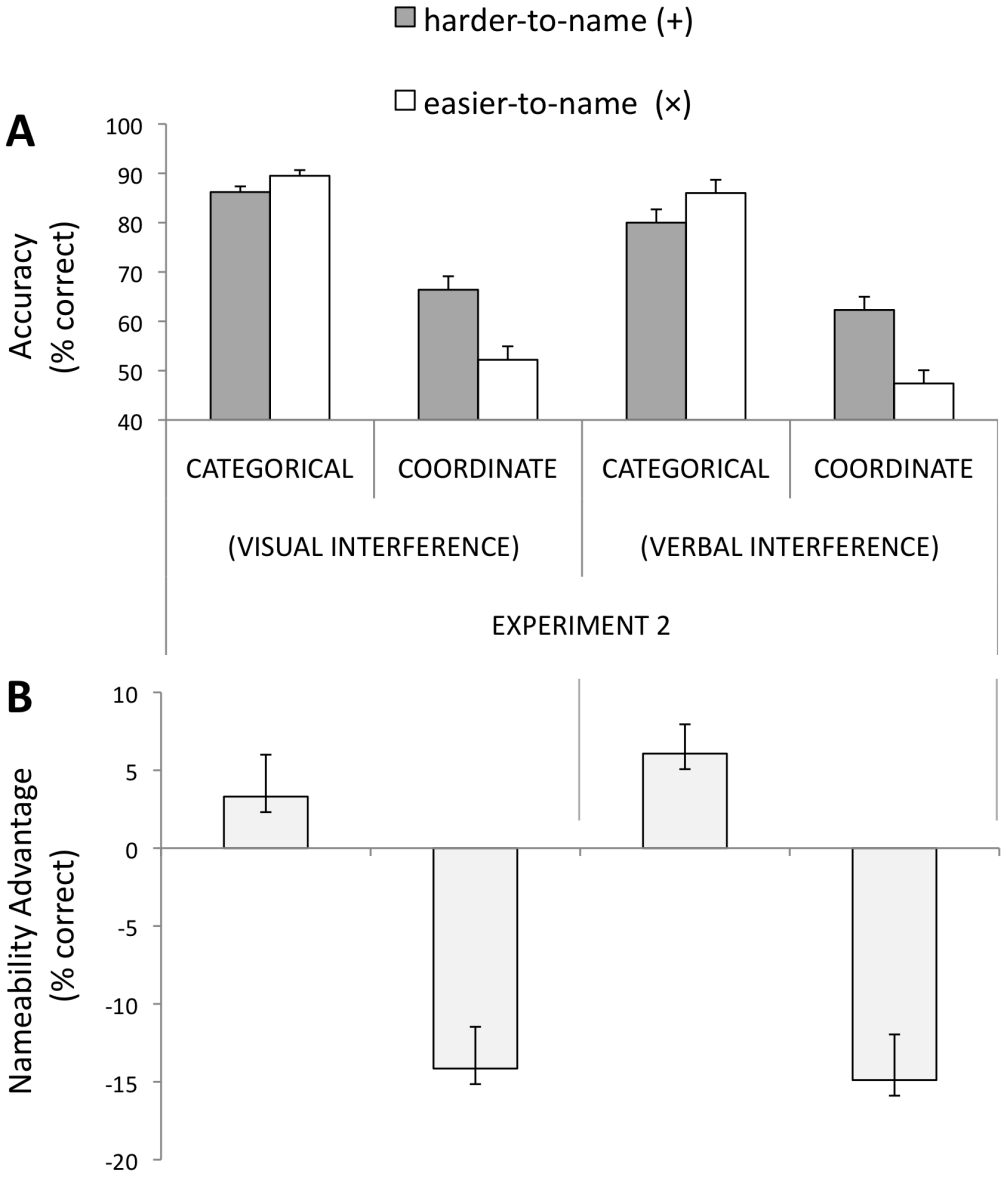
Accuracy results for Experiment 2 (visual vs. verbal interference). (A) Overall % accuracy for all different trials. (B) Nameability advantage (easier-to-name trials minus harder-to-name trials). A positive difference score indicates an advantage on easier-to-name trials; a negative score indicates a disadvantage on easier-to-name trials. Error bars represent 1SE of the mean difference between easier-to-name and harder-to-name trials.

**Accuracy on Main Task**. Unsurprisingly, interference reduced overall accuracy: as revealed by a reliable effect of experiment (Exp. 1 vs. Exp. 2), *F*(1,25) = 6.05, *p* = .015 (cf. Figs. 3 and 4). However, as in Experiment 1, there was a significant main effect of spatial relation-type; overall, participants were more accurate on categorical as compared to coordinate trials, *F*(1, 16) = 19.12, *p*<.0005 (see Figure 4).

Although more errors were made on the visual match/nonmatch task (suggesting it may have been a more challenging interference task), verbal-interference led to poorer overall accuracy on same/different judgments in the main task (mean accuracy  = 68.9%) as compared to performance on same/different judgments during concurrent visual-interference (mean accuracy  = 73.6%) [*F*(1,16) = 6.47, *p* = .02].

Also, as in Experiment 1, we found a significant interaction between nameability (harder-to-name vs. easier-to-name) and spatial relation-type (categorical vs. coordinate), *F*(1,16) = 8.49, *p* = .01] (See Figure 4a). The observed pattern—better performance on easier-to-name “×” trials for categorical judgments as compared to better performance on harder-to-name “+” trials for coordinate judgments—was not affected by interference type [spatial relation-type × nameability × interference type, *F*<1.

Planned t-tests showed a pattern consistent with Experiment 1. With *visual interference*, t-tests show that for categorical relations, performance was 3% more accurate on easier-to-name trials, CI [−0.090, 0.024]—a non-reliable difference, *t*(16) = −1.23, *p*>.20. For coordinate relations, performance was 14.2% less accurate on easier-to-name trials, CI [0.085, 0.198], *t*(16) = 5.28, *p*<.001. With *verbal interference*, performance on categorical trials was boosted by 6% on easier-to-name trials, CI [0.021, 0.101], *t*(16) = 3.22, *p* = .005. For coordinate trials, performance was 14.9% less accurate on easier-to-name trials, *t*(16) = 5.08, *p*<.001, CI [0.087, 0.211] (See Figure 4b).

**Reaction Time**. Participants were faster to respond to categorical (M = 491) compared to coordinate (M = 518) trials, *F*(1, 16) = 7.73, *p*<.013. The effect of nameability was not reliable, *F*<1. As in Experiment 1, there was a reliable spatial relation-type × nameability interaction, *F*(1, 16) = 8.49, *p* = .01, which was also reliable separately for visual-interference and verbal-interference trials. The three-way interaction with interference type included was not reliable, *F*(1, 16) = 2.63, *p* = .13. Planned comparisons for nameability effects on coordinate and categorical trials showed a very similar pattern to Experiment 1. The three-way interaction between relation-type, nameability, and experiment (Exp. 1 vs. Exp. 2) was not reliable, *F*<1. (Nor was this interaction reliable comparing Exp. 1 to the two interference conditions of Exp. 1 separately.)

### 2.3. Discussion

Verbal interference affected overall accuracy on the same/different task more than visual interference, despite the arguably greater demand characteristics of the visual interference task. This finding suggests that language may play some *general* role in the perception of spatial categories. However, we did not find any *selective* effects of verbal interference on discrimination performance for either accuracy or RTs. That is, there was no evidence of a reduction in the difference in accuracy/RTs on the easier-to-name (x) and harder-to-name (+) trials, nor an interaction with trial-type (categorical/coordinate). This pattern of results is not consistent with the prediction that performance on the categorical trials and/or easier-to-name trials is critically dependent on the online recruitment of verbal labels.

## Experiment 3 (Verbal Training)

An alternative to down-regulating the involvement of language via verbal interference, is *up*-regulating the involvement of specific labels via a short training session. Requiring participants to explicitly associate locations with distinct labels strengthens existing associations and tends to exaggerate categorical effects on perception (e.g., see Exp. 3 in [25]). In the present study, Experiment 3 examined whether *training* participants to associate particular meaningful verbal labels with the spatial categories used in Experiment 1 influenced performance on the same/different task immediately following the training session. We hypothesized that if the interaction we observed in Experiment 1 stems from the automatic involvement of category labels, strengthening the association between labels and particular spatial locations should increase the differences between easier-to-name (x) and harder-to-name (+) trials observed in Experiment 1.

Experiment 3 was designed to investigate how explicitly associating four common verbal labels (*above, below, left*, and *right*) with the eight spatial categories—corresponding to the harder-to-name (+) and easier-to-name (×) quadrants used in Experiment 1—affects categorical and coordinate discrimination performance.

### 3.1. Methods

#### 3.1.1 Participants

Fourteen adults participated for pay or course credit (8 women; median age  = 22). All participants were right-handed, native English speakers.

#### 3.1.2. Procedure and Materials

Before performing the same/different judgment task, participants were trained to explicitly associate the eight spatial regions from Experiment 1 with familiar spatial category labels. The session comprised 160 training trials and lasted approximately five minutes. On each training trial participants heard one of four verbal labels (the words above, below, left, or right) followed by the presentation of a red dot in some relation to a central fixation point. The dot appeared in one of the eight positions used in Experiment 1 (see Figure 2C). Importantly, during training participants saw only the origin dot and probe dot: no grid was present. Thus, any partitioning of the space was implicit, being imposed by the participant. Participants were instructed to press the spacebar if the label correctly labeled the spatial relation (80% of trials), and make no response if the label was invalid (20% of trials). The trial ended when a response was made or after 1 s. Correct responses were followed by a ‘bleep’ sound; incorrect responses were followed by a ‘buzz’ sound.

This paradigm trained participants to explicitly classify all eight positions (i.e. the locations of the probes for the × and + partition in Experiment 1) into 4 lexicalized categories. This meant that each of the four positions corresponding to the “×” partition was valid for a single label (*above*, *below*, *left*, or *right*) whereas each of the four positions corresponding to the “+” partition was valid for two labels (*above* and *right*, *above* and *left*, *below* and *right*, or *below* and *left*). (Figure 2C).

After training, participants performed the identical same/different task used in Experiment 1. We hypothesized that if discrete verbal labels played a role in generating the original pattern of results, explicit verbal training would *amplify* the effects by exerting a selective influence on spatial perception between trial types. That is, for the easier-to-name (×) trials, categorical judgments would be predicted to become easier, and the coordinate judgments more difficult as compared to the identical judgments without prior training (as in Experiment 1). Conversely, for harder-to-name (+) trials, categorical judgments should become more difficult and the coordinate judgments easier. In other words, training with verbal labels would further discretize the easier-to-name “×” quadrants while making harder-to-name “+” quadrants more ambiguous (See Figure 2C).

### 3.2. Results and Discussion

**Training Task**. RT performance on training trials confirmed that “×” quadrants depicted more prototypical spatial concepts; participants were much faster in matching an auditory verbal label (*above, below, left*, and *right*) to a dot presented in the four locations corresponding to the “×” quadrants compared to the four locations corresponding to the “+” quadrants from Experiment 1 [*M*_×_  = 399 ms, *M*_+_  = 449 ms, *t*(13) = 7.63, *p*<.0005]. This confirms our initial assumption that the “×” partition demarcates categories that are indeed “easier-to-name” than quadrants demarcated by the “harder-to-name” “+” partition.

**Figure 5:**
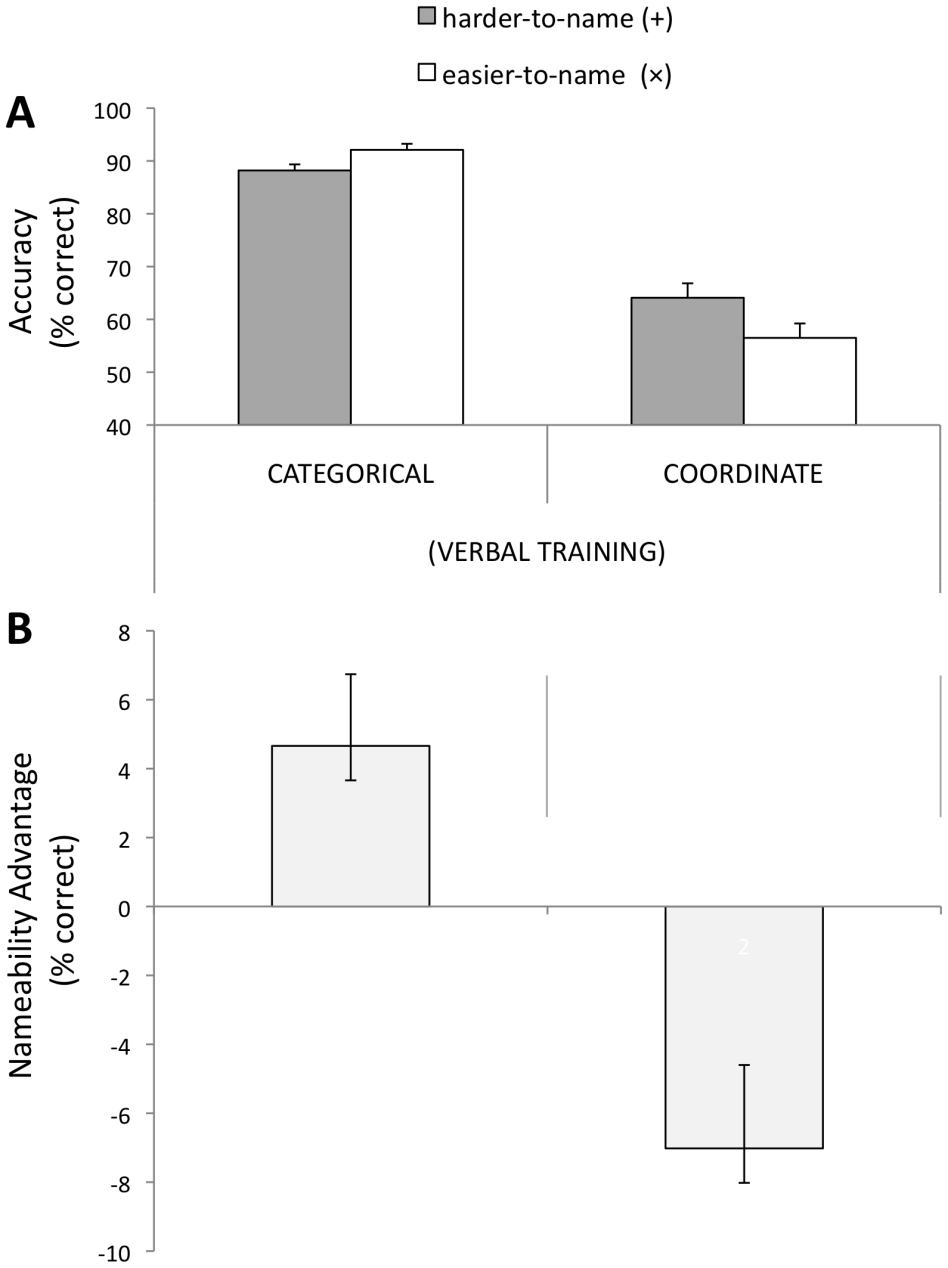
Accuracy results for Experiment 3 (verbal training). (A) Overall % accuracy for all different trials. (B) Nameability advantage (easier-to-name trials minus harder-to-name trials). A positive difference score indicates an advantage on easier-to-name trials; a negative score indicates a disadvantage on easier-to-name trials. Error bars represent 1SE of the mean difference between easier-to-name and harder-to-name trials.

**Accuracy on Main Task**. As evident in Fig. 5b there was a significant main effect of spatial relation-type on accuracy. As in the previous studies, participants were more accurate on categorical than coordinate trials, *F*(1,13) = 81.92, *p*<.0005. The main effect of nameability was not significant. Overall accuracy did not differ significantly between Experiments 1 and 3 (*F*<1).

Planned t-tests show that for categorical relations, performance was more accurate on easier-to-name trials by 4.6%, CI [0.002, 0.092], *t*(13) = 2.24, *p* = .04. For coordinate relations, performance was more accurate on harder-to-name trials by 7.0%, CI [0.018, 0.123], *t*(13) = 2.90, *p* = .01.

As in Experiments 1 and 2, participants in Experiment 3 were more accurate in making categorical judgments for easier-to-name trials relative to harder-to-name trials, while more accurate in making coordinate judgments for harder-to-name trials, relative to easier-to-name trials [Accuracy: *F*(1,13) = 12.07, *p* = .004]. As evident from comparing Figures 3 and 5, verbal training did not meaningfully alter the discrimination profile. The three-way interaction between spatial-relation type, nameability, and experiment (Exp. 1 vs Exp. 3) was entirely absent for accuracy, *F* = 0.0.

**Reaction Time**. Overall, the RTs in the present experiment were faster than in Experiment 1 (M_NO TRAINING_  = 603 ms, M_VERBAL TRAINING_  = 498 ms, [*F*(1,22)  = 4.44, *p* = .047]. The decrease in RTs is most parsimoniously explained as a practice effect resulting from increased exposure to the spatial locations during training. In addition to the main effect of training on RT, there was, as in Experiments 1 and 2, a very reliable categorical-trial advantage (M_CAT_  = 481 ms; M_COOR_  = 516 ms), *F*(1,13) = 18.88, *p* = .001. The main effect of nameability was not significant. The spatial relation type × nameability interaction for RT was in the same direction as in the earlier experiments, but not reliable, *F*(1,13) = 1.18, *p*>.20. The three-way interaction between spatial-relation type, nameability, and experiment (Exp. 1 vs. Exp. 3) was marginal, *F*(1, 66) = 3.77, *p* = .06.

Together, Experiments 2 and 3 suggest that online down-regulation using verbal interference (Exp. 2) or up-regulation using verbal training (Exp. 3) does not alter the degree to which lexicalized spatial categories selectively influence the discrimination of categorical and coordinate spatial relations.

## General Discussion

To investigate top-down effects of spatial categories on perception, we developed a sensitive task capable of detecting the influence of nonverbal semantics on spatial discrimination. Our basic finding is that the discrimination of spatial relations is strongly influenced by spatial categories. Compared to categories associated with multiple lexical items (above-left, etc.), categories associated with simple labels (above, below, right, left) partition space more effectively as evidenced by superior between-category discrimination (categorical trials) and poorer within-category discrimination (coordinate trials). This pattern of results proved to be remarkably robust, resisting the influence of manipulations intended to either disrupt or enhance the influence of language: down-regulating via verbal interference (Exp. 2) and up-regulating via explicit label training (Exp. 3).

One interpretation of the pattern of effects observed in Experiment 1 is that verbal representations of discrete prepositions guided visual perception, facilitating between-category judgments (required by the categorical trials) and interfered with within-category judgments (required by the coordinate trials). While it remains possible that even more sensitive perceptual and interference manipulations (or neuroscience methods) could one day provide evidence that the nameability x spatial information-type interaction reported here is significantly mediated by verbal labels, the lack of selective effects for *both* interference and training manipulations in Experiments 2 and 3 argues against such an interpretation. At the very least, Experiments 2 and 3 reflect a “good effort” to test the hypothesis that the original effect was modulated on-line by verbal labels [29].

The overall pattern of results suggests that spatial categories lexicalized by English prepositions are deeply entrenched and influence the perception of *both* categorical and coordinate spatial information. While these spatial categories are marked in language, we found little evidence to suggest that the observed selective effects for categorical and coordinate processing are under online control of language. This pattern may indicate that perceptual systems are predisposed towards marking some critical spatial boundaries prior to language exposure (like those which distinguish between vertical and horizontal boundaries/projections), and that verbal labels reflect these spatial categories. This interpretation does not rule out the idea that our habits of using particular prepositions and attending to the spatial relations they denote produces biases in spatial categorization [30]. Although we cannot currently distinguish between these two non-mutually exclusive accounts, our data suggest that the influence of spatial categories is resistant to online perturbations of verbal labels.

Furthermore, we found that lexicalized categories predicted performance in a perceptual task that did not always require participants to make a categorical discrimination. That is, the relative difficulty on easier-to-name coordinate trials is especially notable because it provides evidence that lexicalizable categorical information more than ambiguously lexicalizable categorical information influences the perception of continuous, metric spatial information. This finding suggests that high-level categorical information penetrates perceptual representations [25].

More speculatively, these data suggest that mental structures like *schemas* may play a role in representing meaningful patterns of basic spatial relations [31], [32], [33], [34], [35], [36], [37]. In theory, schemas capture an intermediate level of representational structure that is neither perceptual nor verbal in nature. As such, schemas lack both the rich detail of percepts, and the symbolic properties of words, while encoding the necessary content for representing primitive relational semantics. What distinguishes schemas from percepts is that they are *meaningful*, encoding the basic semantics contained within asymmetrical figure-ground relations. But a schematic level of representation is also distinguishable from language in that it is relatively more *analog*. Whereas words are digital, with their relations to the entities they denote being largely arbitrary, schematic representations maintain the generalized topographic structure of the percept. With respect to the current study, a schematic representation (or *image schema*) can serve the purpose of representing lexicalized meaning nondigitally. While the theoretical construct of an image schema is widely influential in cognitive linguistics and conceptual metaphor theory, few experimental studies have lent them empirical support. In line with this theoretical construct, the present data suggests that meaningful analog spatial representations exert an influence on perception, and are not easily modulated by verbal interference or training. Consistent with the findings of two recent studies, the present data also suggest that meaningful schematic spatial representations [32] exert influence on low-level spatial relational processing in a manner that relatively digital representations alone (like words) cannot [38]. Space may be special in this respect. That is, while space may play an important role in grounding semantics (in an “embodied” sense) it may simultaneously be more resistant to Whorfian effects *because* it serves such a fundamental role structuring our larger, more abstract conceptual system in an analog manner.

## Conclusions

Kosslyn [14] proposed a hemispheric bias for processing categorical and coordinate spatial information. He argued that the neuroanatomical division of labor that predisposes the left hemisphere for categorical information and the right hemisphere for coordinate information is the result of a long evolutionary history. Kosslyn [14] first speculated that cortical specializations for categorical processing in the left hemisphere, and coordinate processing in the right, was the evolutionary result of prior hemispheric specializations for speech. Later, Kosslyn [18] revised his theory, hypothesizing that low-level perceptual biases in left hemisphere structures important for abstraction (and categorization) served as a precursor for the development of language in proximate cortical areas. Although the present study did not investigate lateralization, the data is at least consistent with a third possible hypothesis suggested by previous research [16]. This recent patient study using voxel lesion symptom mapping provided evidence that the right hemisphere exhibits an analog bias in representing the meaning of basic spatial categories. One possibility is that within the right hemisphere, analog representations of basic spatial categories exert a modulating effect on the perception of coordinate spatial relations independent of relatively digital representations processed in the left hemisphere.

Nearly twenty years after coining the distinction between categorical and coordinate spatial information Kosslyn [39] wrote, “I would not be surprised if the distinction between categorical and coordinate spatial relations provides insight into how linguistic categories bridge to perceptual representations" (p. 1523). While the present data suggests a limited role for online verbal labels in modulating spatial perception, it also presents evidence that meaningful and lexicalized–but relatively analog– representations of spatial categories can bias perceptual judgments about metric spatial information. Still, developing a better understanding of how long-term experience with naming might influence both concept formation and perceptual guidance, for these and other spatial categories, remains a rich ground for further investigation. The present data show an interaction between lexicalized spatial categories and the processing of lower-level coordinate spatial information providing insight into the continuity across perception and conception—what Talmy [34] called *ception*. Our findings are consistent with the general view that verbal, conceptual, and perceptual representations share a parallel structure [40] and shed further light on the organization of a neural system capable of representing the most basic meanings on a digital-analog continuum.
